# Genome-Scale Phylogenetic Evidence Supports the Synonymy of *Lasiodiplodia brasiliensis* with *Lasiodiplodia theobromae*

**DOI:** 10.3390/jof12040270

**Published:** 2026-04-08

**Authors:** Celynne Ocampo-Padilla, Yoshiki Takata, Shunsuke Nozawa, Yui Harada, Katsuhiko Ando, Kyoko Watanabe

**Affiliations:** 1 Graduate School of Agriculture, Tamagawa University, Tamagawa-Gakuen 6-1-1, Machida 194-8610, Japan; celynnepadilla@clsu.edu.ph (C.O.-P.); t0h522221@stu.tamagawa.ac.jp (Y.H.); 2 Department of Crop Protection, College of Agriculture, Central Luzon State University, Science City of Muñoz 3120, Nueva Ecija, Philippines; 3 College of Agriculture, Tamagawa University, Tamagawa-Gakuen 6-1-1, Machida 194-8610, Japan; mandy3takata@gmail.com (Y.T.); nozao802@gmail.com (S.N.); scutisporus@gmail.com (K.A.)

**Keywords:** *Lasiodiplodia*, integrative taxonomy, genome-scale phylogeny, species delimitation, Botryosphaeriaceae

## Abstract

The genus *Lasiodiplodia* includes numerous plant-pathogenic species whose delimitation is complicated by overlapping morphological traits and limited resolution of common genetic markers. *Lasiodiplodia brasiliensis* was described as a species closely related to *L. theobromae*; however, its taxonomic status remains controversial. In this study, we re-evaluated the species boundaries between *L. theobromae* and *L. brasiliensis* using an integrative approach that combined multilocus and genome-scale phylogenetic analyses with morphological comparisons. Multilocus phylogenetic analyses based on ITS, *tef1-α*, *tub2*, and *rpb2* revealed an unresolved relationship between the two taxa. The *L. theobromae* clade had low bootstrap support, whereas the ancestral node connecting both species had high support. In contrast, genome-scale phylogenetic analysis using hundreds of single-copy orthologous genes strongly supported a single monophyletic clade encompassing isolates assigned to both *L. theobromae* and *L. brasiliensis*. Morphological analyses further revealed that conidial dimensions and other diagnostic characteristics largely overlapped between the two taxa, rendering them unreliable criteria for species separation. Considering the combined molecular and morphological evidence, our results support treating *L. brasiliensis* as a synonym of *L. theobromae*. Clarifying species boundaries within this group helps stabilize the taxonomy of *Lasiodiplodia* and provides a reliable foundation for accurate pathogen identification and disease management.

## 1. Introduction

*Lasiodiplodia* is a genus belonging to the family Botryosphaeriaceae (order Botryosphaeriales, phylum Ascomycota). Although once considered monotypic, the genus now comprises more than 80 described species, with approximately 70 species delimited based on morphological characteristics and molecular data [1,2]. However, many *Lasiodiplodia* species exhibit overlapping morphological traits, making identification based solely on morphology unreliable [3]. Consequently, molecular approaches have become essential for accurate species identification within the genus.

Accurate identification of plant pathogens is critical for effective disease management in agriculture. In the Philippines, species of *Lasiodiplodia* have recently been recognized as important pathogens of cacao (*Theobroma cacao* L.), a high-value crop for which species-specific management strategies have not yet been established. During investigations of cacao diseases, two species of *Lasiodiplodia*, *L. theobromae* (Pat.) Griffon & Maubl. and *L. brasiliensis* M.S.B. Netto, M.W. Marques & A.J.L. Phillips, were consistently isolated. These taxa are morphologically indistinguishable and form a single clade in multilocus phylogenetic analyses, raising questions regarding their taxonomic separation.

Resolving species boundaries in *Lasiodiplodia* is challenging. This is due to limited phylogenetic resolution from commonly used loci and high levels of intraspecific variation [2]. Particularly, species described based on subtle morphological differences and limited sampling may represent conspecific lineages rather than distinct taxa. Such taxonomic ambiguity is not only a systematic concern but also hampers accurate pathogen identification and communication in plant disease research and management.

*L. theobromae* is one of the most frequently reported species in the genus and occurs across a wide range of hosts and geographic regions [3,4]. In contrast, *L. brasiliensis* was described more recently [5,6,7] and has been reported from a narrower host range. Previous phylogenetic studies have shown that these two taxa are closely related and often unresolved in multilocus analyses, suggesting their potential conspecificity. However, a comprehensive reassessment incorporating multiple lines of evidence remains limited. Therefore, we addressed this gap by applying an integrative taxonomic approach, combining multilocus and genome-scale phylogenetic analyses and morphological comparisons to evaluate the taxonomic relationship between *L. theobromae* and *L. brasiliensis* isolated from cacao in the Philippines. By clarifying species boundaries within this complex, our study aims to stabilize *Lasiodiplodia* taxonomy and improve the accuracy of pathogen identification.

## 2. Materials and Methods

### 2.1. Sampling and Fungal Isolation

Cacao diseases, including leaf blight, vascular streak, and pod rot, as well as asymptomatic flowers, were collected from cacao fields in Nueva Ecija, Pangasinan, and Davao, Philippines. Sample tissues were cut into small pieces (approximately 5 mm^2^), surface-sterilized with 1% sodium hypochlorite for 2 min, rinsed twice in sterile distilled water, and dried on sterilized paper. The tissues were plated onto water agar (WA) and incubated at 25 °C for 7 days. Hyphae emerging from the tissues were transferred onto potato dextrose agar (PDA; Eiken Chemical Co., Ltd., Tokyo, Japan) plates and incubated for 3–14 days under ambient light near a window to induce sporulation. Fungi exhibiting diagnostic characteristics of the genus *Lasiodiplodia*, such as septate conidia with longitudinal striations [8], were selected for further experiments. Monocultures were obtained by single-spore isolation and maintained on one-fifth-strength PDA. Each isolate was preserved in 10% glycerol and stored at −80 °C at Tamagawa University, Tokyo, Japan.

### 2.2. DNA Extraction and Amplification

Genomic DNA was extracted from 4–7-day-old cultures of each *Lasiodiplodia* isolate using the cetyltrimethylammonium bromide (CTAB) method [9]. Four loci were targeted for multilocus analysis: the rDNA internal transcribed spacer region (ITS1, 5.8S rDNA, ITS2), β-tubulin (*tub2*), RNA polymerase subunit II (*rpb2*), and translation elongation factor 1-alpha (*tef1-α*). These loci were amplified using the primer pairs ITS4/ITS5 [10], Bt2a/Bt2b [11] or btLasF/btLasR (this study), rpb2-LasF/rpb2-LasR [12], and EF1-LasioF/EF1-LasioR [13] (Table S1). The primers btLasF/R and EF1-LasioF/R, designed from NGS-derived genomic data of *Lasiodiplodia* spp., were developed to improve amplification efficiency and sequence quality in isolates where previously published primers occasionally produced weak amplification or noisy chromatograms.

PCR reactions were performed in 10 μL volumes, consisting of 1 μL of 10× Taq buffer, 0.8 μL of dNTPs (2.5 mM each), 0.1 μL of each primer, 0.05 μL of Ex Taq polymerase (TaKaRa Ex Taq™, Takara, Shiga, Japan), 7 μL of double-distilled water, and 1 μL of genomic DNA. Specific PCR conditions for each locus are provided in Supplementary Table S2. PCR products were purified using ExoSAP-IT (GE Healthcare, Tokyo, Japan). Cycle sequencing was conducted using the Quantum Dye Terminator Cycle Sequencing Kit (SureFire Biosciences, Sheridan, WY, USA), and DNA sequences were obtained from the FASMAC DNA sequencing service (Kanagawa, Japan). All sequences generated in this study were deposited in the DNA Data Bank of Japan (DDBJ; Supplementary Table S3).

### 2.3. Genome Sequencing and Assembly

For genome-scale analysis, genomic DNA was extracted from 32 strains, including six isolates obtained in this study (TAP23C-1288, TAP23C-1289, TAP24C-0111, TAP24C-0116, TAP24C-0119, and TAP25C-0100), four isolates from a previous study (PH22-014, PH22-060, PH22-080, and PH22-120) [13], 16 strains from the Westerdijk Fungal Biodiversity Institute (WI-KNAW) culture collection, and six strains from the National Agriculture and Food Research Organization (NARO) Genebank (Table 1). Approximately 0.6–0.8 g of dried mycelium was processed using the CTAB method combined with the Maxwell^®^ RSC PureFood GMO and Authentication Kit on a Maxwell^®^ RSC instrument (Promega Corporation, Fitchburg, WI, USA). DNA concentration and quality were assessed using a NanoDrop spectrophotometer (Thermo Fisher Scientific, Waltham, MA, USA) and 1% agarose gel electrophoresis.

Genome sequencing was performed by Gene Nex using Illumina high-throughput sequencing technology (NovaSeq X). Raw sequencing reads were trimmed to remove adapter sequences and low-quality bases using Trimmomatic v0.40, and read quality was evaluated using FastQC v0.11.5 [14]. Genome assemblies were generated using Platanus allee v2.0.2 [15].

### 2.4. Molecular Phylogenetic Analysis

For multilocus phylogenetic analysis, we used sequence data from 62 reference strains of *Lasiodiplodia*, including ex-type strains, and 41 isolates obtained in this study. *Botryosphaeria dothidea* CBS 115476 was used as an outgroup (Supplementary Table S3). Accession numbers for reference strains were obtained from Huda-Shakirah et al. (2022) [16] and our previous study [13]. Sequences were aligned using ClustalW, which was implemented in MEGA v7 [17]. Phylogenetic trees were inferred using neighbor-joining (NJ), maximum likelihood (ML), and maximum parsimony (MP) methods based on concatenated alignments of ITS, *tef1-α*, *tub2*, and *rpb2*. For ML analysis, the Tamura three-parameter model with a gamma distribution (T92 + G) was selected based on the Akaike information criterion. Gaps were considered missing data. Branch support was assessed using bootstrap analysis with 1000 replicates [18].

Genome-scale phylogenetic analysis was performed using genomic data from 64 strains representing 19 species of *Lasiodiplodia*, with *Fusarium graminearum* PH-1 and *Lecanosticta acicola* CBS 871.95 included as outgroup taxa (Table 1). Genome sequences of 32 strains were retrieved from NCBI [19], whereas the remaining 32 genomes were generated in this study. Single-copy orthologs were identified using OrthoFinder v2.5.4 [20] with default parameters. Orthologous sequences were aligned using MAFFT v7.505 [21] and trimmed with trimAl v1.4 [22] using the option −g 10. An NJ tree was inferred from the concatenated alignment using MEGA v11 [23] to provide an overview of the genome-scale relationships. Bootstrap analysis was performed with 100 replicates.

### 2.5. Morphological Analysis

Colony morphology of *L. theobromae* and *L. brasiliensis* was examined on PDA after incubation at 25 °C for 7 days under a 12 h light/12 h dark cycle. Pycnidia were induced on autoclaved cacao leaves and stems placed on WA plates and incubated at 25 °C under ambient light conditions for 7–21 days. Fungal structures were observed and photographed using a Leica Wild Stereo Microscope MDG-17 (Leica Microsystems GmbH, Wetzlar, Germany). Conidia (n = 30 or 50), conidiogenous cells (n = 30), and paraphyses (n = 30) were examined using an Olympus BX51 light microscope (Olympus, Tokyo, Japan) and measured using ImageJ v1.53.

Differences in conidial dimensions among isolates were statistically evaluated using Welch’s one-way analysis of variance (Welch ANOVA) owing to unequal sample sizes among isolates. Test statistics were calculated using the Welch–Satterthwaite approximation, and significance was assessed at α = 0.05. Pairwise comparisons were performed using the Games–Howell post hoc test, and isolates were grouped accordingly, with identical letters indicating no significant differences. All statistical analyses were conducted in R v4.5.2 [24] using the rstatix [25] and multcompView packages [26].

To determine optimal growth temperatures, 5-mm-diameter mycelial plugs from the margins of 4-day-old colonies were transferred onto PDA plates and incubated at 4, 10, 15, 20, 25, 30, and 35 °C. Colony diameter was measured after 24 h of incubation, with five replicate plates per isolate at each temperature.

## 3. Results

### 3.1. Sampling and Fungal Isolation

We obtained 41 isolates of *Lasiodiplodia* from three sampling sites: 21 from Pangasinan, 19 from Davao, and one from Nueva Ecija. Among these, 12 isolates were obtained from symptomatic leaves, 17 from symptomatic stems, four from symptomatic flowers, four from asymptomatic flowers, three from symptomatic pods, and one from leaf litter (Supplementary Table S4).

### 3.2. Molecular Phylogenetic Analysis

For multilocus phylogenetic analysis, the relationships among the 41 *Lasiodiplodia* isolates were inferred using NJ, ML, and MP methods based on a concatenated dataset of four loci: ITS (495 bp), rpb2 (465 bp), tef1-α (408 bp), and tub2 (455 bp).

**Figure 1:**
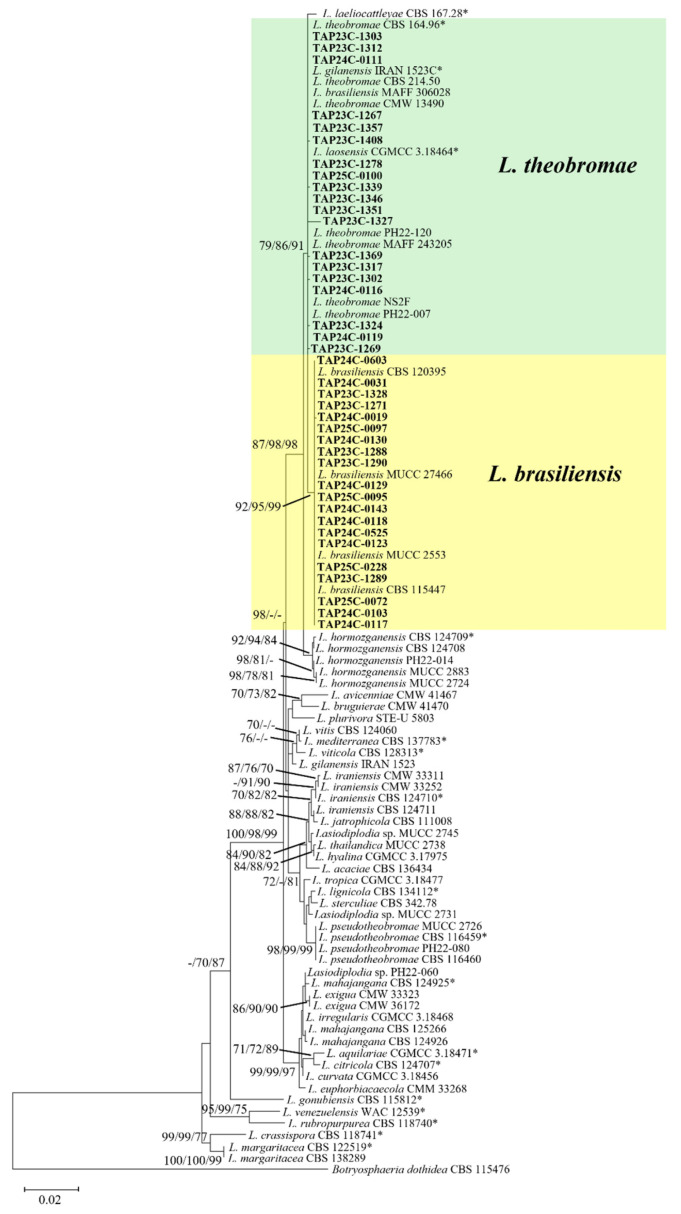
Maximum likelihood (ML) phylogenetic tree of *Lasiodiplodia* spp. based on concatenated sequences of ITS, *tub2*, *tef1-α*, and *rpb2.* Bootstrap support values ≥ 70% from ML, neighbor-joining (NJ), and maximum parsimony (MP) analyses are shown above or below the corresponding nodes in the order ML/NJ/MP. Ex-type strains are indicated by asterisk. Isolates obtained in this study are shown in bold.

Among the 41 isolates analyzed, 21 clustered with *L. theobromae*, with relatively low bootstrap support (ML/NJ/MP = 69/39/0), whereas 20 clustered with *L. brasiliensis* with high bootstrap support (ML/NJ/MP = 95/94/99). The node uniting these two taxa was supported by bootstrap values of 86/81/92 (ML/NJ/MP; Figure 1).

**Figure 2:**
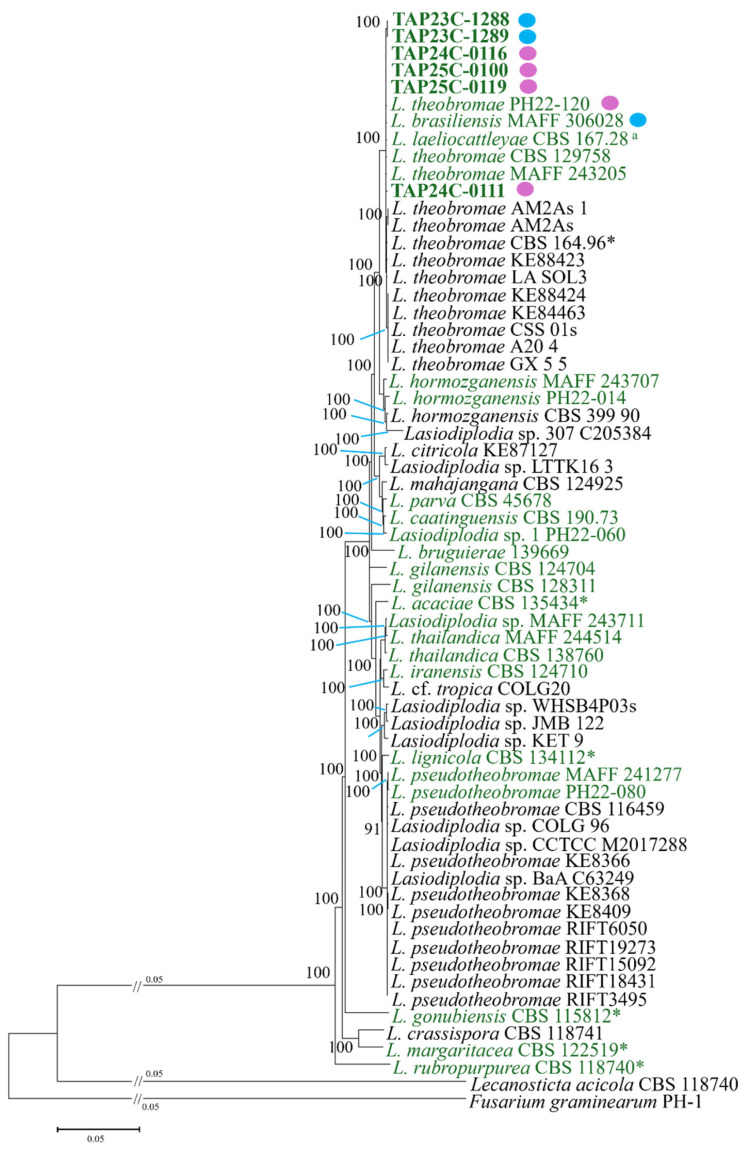
Genome-scale phylogenetic tree of *Lasiodiplodia* spp. inferred using the neighbor-joining (NJ) method. Isolates newly obtained in this study are indicated in bold green, whereas isolates for which genome data were generated in this study are written in green. Ex-type strains are indicated by an asterisk. The ex-type isolate corresponding to the previously named *L. laeliocattleyae* is marked as a superscript “a”. Pink and blue circles indicate isolates assigned to the *L. theobromae* and *L. brasiliensis* clades, respectively, based on multilocus phylogenetic analysis.

Genome-scale phylogenetic analysis was conducted to further resolve the relationship between *L. theobromae* and *L. brasiliensis*. The concatenated alignment of 663 predicted single-copy orthologous genes comprised 645,633 bp, including 389,189 variable and parsimony-informative sites. In the resulting NJ tree, isolates identified as *L. theobromae* and *L. brasiliensis* clustered within a single highly supported clade (Figure 2). The seven isolates assigned in this study (TAP24C-0116, TAP24C-0111, TAP25C-0100, TAP25C-0119, PH22-120, TAP23C-1288, and TAP23C-1289) were distributed within this lineage, consistent with the multilocus phylogeny (Figure 1). Notably, the reference isolate *L. brasiliensis* MAFF 306028 was nested within the *L. theobromae* clade.

### 3.3. Morphological Analysis

**Figure 3:**
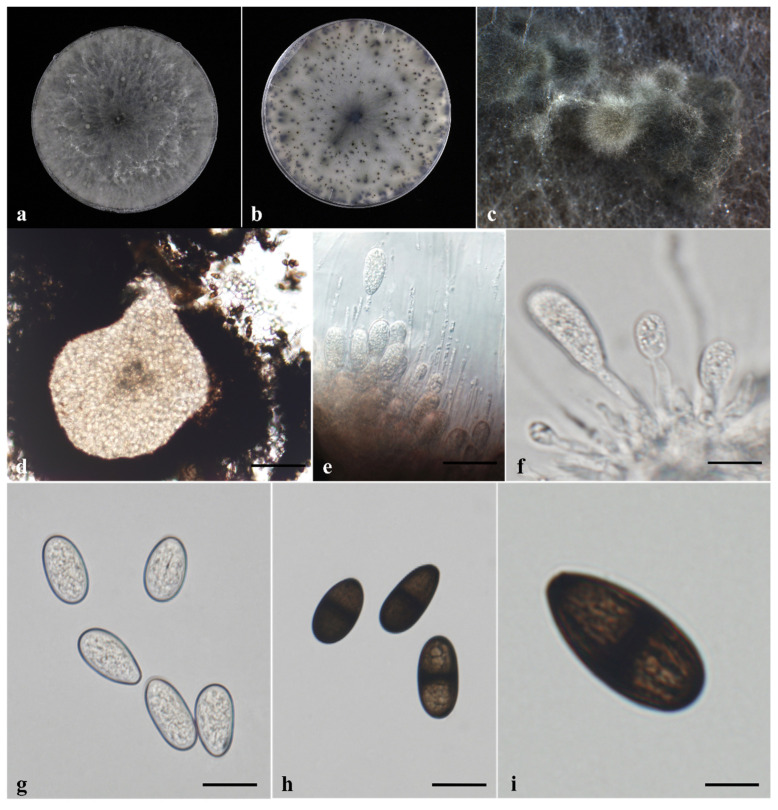
Morphological characteristics of *Lasiodiplodia theobromae* PH22-120. (**a**) Upper view and (**b**) reverse view of colony growth on potato dextrose agar (PDA) after 7 days under a 12 h light/12 h dark photoperiod; (**c**) conidiomata formed on cacao stem on water agar (WA); (**d**) conidiomata; (**e**) hyaline paraphyses formed among conidiogenous cells; (**f**) conidiogenous cells producing conidia; (**g**) hyaline, immature, smooth-walled conidia; (**h**) dark, mature, septate conidia; (**i**) mature conidia at different focal planes showing longitudinal striations. Scale bars: (**d**–**h**) = 20 μm.

**Figure 4:**
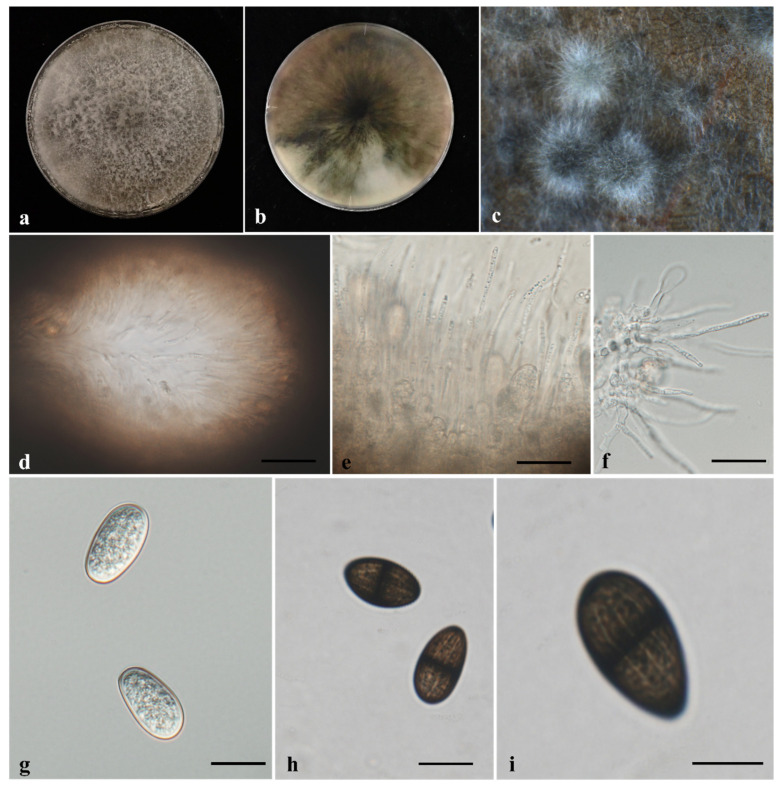
Morphological characteristics of *Lasiodiplodia brasiliensis* TAP23C-1289. (**a**) Upper view and (**b**) reverse view of colony growth on potato dextrose agar (PDA) after 7 days under 12 h light/12 h dark photoperiod; (**c**) conidiomata formed on cacao stem on water agar (WA); (**d**) conidiomata; (**e**) hyaline paraphyses formed between conidiogenous cells; (**f**) conidiogenous cells producing conidia; (**g**) hyaline immature smooth-walled conidia; (**h**) dark mature septate conidia; (**i**) mature conidia at different focal plane showing longitudinal striations. Scale bars: (**d**–**h**) = 20 μm.

Overall, the isolates examined in this study exhibited morphological characteristics consistent with those of *Lasiodiplodia*. Pycnidia formed on sterilized cacao leaves and stems placed on PDA were aggregated, globose, gray to black, and covered with hyphae (Figure 3c and Figure 4c). Paraphyses in the pycnidial conidiomata were hyaline, septate or aseptate, smooth-walled, and had rounded apices (Figure 3e and Figure 4e). Conidiogenous cells were hyaline, smooth, thin-walled, cylindrical, and holoblastic (Figure 3f and Figure 4f). Both immature and mature conidia were observed; conidia were subovoid to ellipsoid-ovoid in shape, with a rounded apex and a truncated base. Immature conidia were hyaline, aseptate, and smooth-walled, containing granular contents (Figure 3g and Figure 4g), whereas mature conidia were light to dark brown, one-septate with a median septum, exhibited longitudinal striations, and were widest at the center (Figure 3h,i and Figure 4h,i). Conidial dimensions are summarized in Table 2.

Furthermore, conidial dimensions were measured and statistically analyzed using R version 4.5.2 [23]. Welch ANOVA revealed highly significant differences among isolates for both conidial length (F = 41, *p* < 0.001) and width (F = 41, *p* < 0.001), indicating substantial variation in conidial size among individual isolates. Games–Howell post hoc tests further identified isolate pairs within each phylogenetic clade that differed significantly in mean conidial length and width (Table 2). In contrast, most isolate pairs between the two clades defined in the ML phylogenetic tree (Figure 1) did not show significant differences in conidial dimensions.

Comparison of conidial sizes of isolates assigned to the *L. theobromae* and *L. brasiliensis* clades with published descriptions of the ex-type strain of *L. theobromae* (24.49–27.49 × 13.3–14.79 μm) [27] and *L. brasiliensis* (22.7–29.2 × 11.7–17.0 μm) [7] revealed overlapping size ranges, including isolates forming the smallest and largest conidia within the two clades (Table 2). Measurements of paraphyses and conidiogenous cells were (22.5–)38.8–49.7(–66.0) × (1.5–)2.3–2.8(–3.7) μm (mean ± SD: 45.1 ± 7.6 × 2.6 ± 0.4; L/W = 17.2) and (5.3–)9.3–12.0(–16.0) × (1.0–)2.9–4.1(–6.0) μm (mean ± SD: 10.8 ± 2.1 × 3.5 ± 0.8; L/W = 3.1), respectively. Direct comparisons of these measurements with those of the ex-type strain of *L. theobromae* were not possible due to the absence of corresponding morphological data in previous studies.

Colony growth of *L. theobromae* and *L. brasiliensis* was examined on PDA for 7 days. Colonies of both species produced aerial mycelium that was initially white and gradually became light gray to gray at 25 °C under a 12 h light/12 h dark photoperiod. Under continuous darkness, colonies grown at 30 °C reached the edge of 90-mm Petri dishes within 2 days, indicating this temperature as optimal for mycelial growth. The minimum and maximum growth temperatures observed were 10 and 35 °C, respectively.

## 4. Discussion

The genus *Lasiodiplodia* has undergone a rapid increase in the number of recognized species in recent years. However, species delimitation within the genus remains challenging owing to extensive overlap in morphological characters and the frequent occurrence of cryptic species complexes. Consequently, identification based solely on traditional morphological criteria is often unreliable. Although integrative approaches combining morphology with multilocus phylogenetic analyses are now widely used [3,28], the taxonomic boundaries between *L. theobromae* and *L. brasiliensis* have remained unclear since *L. brasiliensis* was described as a closely related taxon.

In the present study, we re-evaluated the species boundaries between *L. theobromae* and *L. brasiliensis* using multilocus and genome-scale phylogenetic analyses in combination with conidial morphology. In the multilocus phylogeny, the *L. brasiliensis* clade was well supported, whereas the *L. theobromae* clade showed low bootstrap support. Simultaneously, the ancestral node uniting the two taxa received high support, indicating unresolved phylogenetic relationships. Taken together, these results provide limited evidence for treating *L. theobromae* and *L. brasiliensis* as distinct species based solely on multilocus data. This interpretation is consistent with that of Ko et al. (2023) [2], who suggested that *L. brasiliensis* represents intraspecific variation within *L. theobromae* rather than a separate evolutionary lineage.

Although computational species delimitation methods are often used to explore potential species boundaries in multilocus datasets, the objective of this study was to reassess the taxonomic status of *L. brasiliensis* relative to *L. theobromae* using genome-scale phylogenomic data and morphological comparisons. Genome-scale phylogenomic analysis provided additional resolution for evaluating the relationship between *L. theobromae* and *L. brasiliensis*. The phylogeny inferred from hundreds of single-copy orthologous loci consistently recovered isolates previously identified as *L. theobromae* and *L. brasiliensis* within a single well-supported clade, within which, isolates assigned to the two taxa were intermingled. In addition, no consistent morphological differences were observed between these taxa. Taken together, these results do not support their recognition as separate species. Therefore, *L. brasiliensis* is best interpreted as a synonym of *L. theobromae*. Similar patterns have been reported in fungi, where genome-scale or multilocus phylogenomic datasets provide greater resolution for evaluating species boundaries and evolutionary relationships than analyses based on a small number of loci [29].

Further support comes from the phylogenetic placement of key reference strains. Strain MAFF 306028, originally identified as *L. theobromae* by Sato et al. (2016) [30] and later reassigned to *L. brasiliensis* by Hattori et al. (2023) [31], consistently clustered with *L. theobromae* in both multilocus and genome-scale analyses in the present study. Similarly, strain CBS 167.28, the ex-type strain of *L. laeliocattleyae*, which has been reported as closely related to *L. theobromae* in multilocus analyses, also formed a monophyletic group with *L. theobromae* in the genome-scale phylogeny. These findings suggest that species boundaries within the *L. theobromae* complex have been inconsistently interpreted and remain unstable when based on single- or few-locus datasets compared to genome-scale analysis.

Morphological evidence further supports the lack of a clear separation between *L. theobromae* and *L. brasiliensis.* Conidial dimensions measured in this study showed no significant differences between isolates assigned to the two clades, indicating that conidial size alone cannot reliably distinguish them. The original description of *L. brasiliensis* relied on morphological characteristics that largely overlap with those of *L. theobromae* and lacked strong diagnostic features [5,7,32].

Taken together, the combined multilocus, genome-scale phylogenetic, and morphological evidence supports the conclusion that *L. brasiliensis* does not represent a species distinct from *L. theobromae*. Accordingly, and following the principle of nomenclatural priority, *L. brasiliensis* is best treated as a synonym of *L. theobromae*.

Despite the strong concordance among multilocus, genome-scale phylogenetic, and morphological data, certain limitations to this study should be acknowledged. The ex-type strain of *L. brasiliensis* (CMM 4015) was not available for analysis, and genome sequence data remain unavailable for several species within the genus *Lasiodiplodia*.

Accurate species identification within *Lasiodiplodia* is essential because members of this genus include important plant pathogens that affect a wide range of hosts. Clarifying the taxonomic status of *L. theobromae* and its synonyms provides a more stable framework for understanding disease etiology, host range, and epidemiology, and supports more reliable disease diagnosis and management strategies.

## 5. Conclusions

In conclusion, this study proved that *L. brasiliensis* should be treated as a synonym for *L. theobromae* based on integrated molecular and morphological analyses. Furthermore, genome-scale phylogenetic inference offers high-resolution support, with both taxa forming a strongly supported monophyletic clade with high bootstrap values. The absence of significant differences in spore dimensions among the examined isolates further corroborates that the two taxa represent a single species. The consistency between genomic data and morphological observations provides compelling evidence for synonymizing these taxa. This taxonomic clarification contributes to improved species delimitation within the genus and has important implications for accurate identification and effective management strategies for diseases caused by *L. theobromae.*

## Figures and Tables

**Table 1:** Strains used in genome-scale phylogenetic analysis and their corresponding gene accession numbers.

Species Name	Strain Name	Accession Number
*Lasiodiplodia acaciae*	CBS 136434 *	– ^a^
*L. bruguierae*	CBS 139669	– ^a^
*L. caatinguensis*	CBS 190.73	– ^a^
*L. citricola*	KE87127	JAJNLL01
*L. crassispora*	CBS 118741 *	– ^a^
*L. gilanensis*	CBS 124704	– ^a^
	CBS 128311	– ^a^
*L. gonubiensis*	CBS 115812 *	– ^a^
*L. hormozganensis*	CBS 339.90	JAUJDW01
	MAFF243707	– ^a^
	PH22-014	– ^a^
*L. iranensis*	CCTCC M2017288	JACEOI01
	CBS 124710	– ^a^
*L. laeliocattleyae*	CBS 167.28	– ^a^
*L. lignicola*	CBS 134112	– ^a^
*L. mahajangana*	CBS 124925	– ^a^
*L. margaritacea*	CBS 122519 *	– ^a^
*L. parva*	CBS 456.78	– ^a^
*L. pseudotheobromae*	WHSB4P03s	JAJQJY01
	RIFT6050	JAWMWL01
	RIFT3495	JAWMWM01
	RIFT19273	JAWMWI01
	RIFT18431	JAWMWJ01
	RIFT15092	JAWMWK01
	CBS 116459 *	RHKG01
	BaA C63249	JAMJPI01
	KET9	JAJMYY01
	PH22C-080	– ^a^
	MAFF241277	– ^a^
*L. rubropurpurea*	CBS 118740 *	– ^a^
*L. thailandica*	MAFF244514	– ^a^
	CBS 138760	– ^a^
*L. theobromae*	LA-SOL3	VCHE01
	KE88424	JAJMYP01
	KE84463	JAJMXW01
	KE8366	JAJMYM01
	JMB122	JAJSDP01
	GX.5.5	JAJQJZ01
	CSS-01s	KZ107826
	CBS 164.96 *	RHKF01000148
*L. theobromae*	KE88423	JAJMYQ01
	AM2As	QCYV01
	AM2As_1	NW_023336399
	LTTK16-3	CP141755
	A20-4	WUMH01
	307	VOPK01
	PH22-120	– ^a^
	MAFF 243205	– ^a^
	TAP25C-0100	– ^a^
	TAP24C-0116	– ^a^
	TAP24C-0111	– ^a^
	TAP24C-0119	– ^a^
	CBS 129758	– ^a^
*L. theobromae* (syn. *L. brasiliensis*)	TAP24C-1289	– ^a^
	TAP24C-1288	– ^a^
	MAFF306028	– ^a^
*Lasiodiplodia* sp.	COLG20	VNWV01
*Lasiodiplodia* sp.	COLG96	VNWU01
*Lasiodiplodia* sp.	KE8409	JAJMVG01
*Lasiodiplodia* sp.	KE8368	JAJMYN01
*Lasiodiplodia* sp.	TAP22C-060	– ^a^
*Lasiodiplodia* sp.	MAFF243711	– ^a^
*Fusarium graminearum*	PH-1	CM000574
*Lecanosticta acicola*	CBS 871.95	AWYC02

* indicates ex-type strain. ^a^ indicates strains with genomic data generated in this study

**Table 2:** Conidial size ranges and mean dimensions of isolates of *Lasiodiplodia* classified by clade.

Clade ^a^	Strain	Length (Mean ± sd)	Width (Mean ± sd)
*L. theobromae*	TAP22C-0007	21.1–25.8 (23.5 ± 1.2)a	12.3–18.4 (14.5 ± 1.4)abefghijk
	TAP22C-0120	23–32.5 (27.4 ± 1.9)cdefghij	12.2–18.3 (14.6 ± 1.1)aefghi
	TAP23C-1267	21.3–30.5 (27.2 ± 2.1)cdefghijkl	13.7–17.3 (14.8 ± 0.9)efghj
	TAP23C-1269	20.5–26.9 (24.5 ± 1.5)abm	11.0–16.7 (13.5 ± 1.1)bcd
	TAP23C-1278	24.1–29.0 (26.1 ± 1.2)ejklp	12.8–17.6 (14.8 ± 1.0)efhj
	TAP23C-1302	23.1–28.0 (25.9 ± 1.1)ekp	12.9–16.8 (14.9 ± 1.0)ehj
	TAP23C-1303	24.1–29.7 (26.8 ± 1.4)cdehijkl	10.2–15.7 (13.2 ± 1.1)cd
	TAP23C-1312	21.2–29.4 (25.3 ± 1.3)mp	12.1–17.1 (14.0 ± 1.1)abcfgi
	TAP23C-1317	23.9–29.9 (27 ± 1.6)cdeghijkl	11.2–16.8 (14.3 ± 1.4)abcdefghi
	TAP23C-1324	23.1–30.0 (27.3 ± 1.5)cdghi	11.5–16.1 (14.0 ± 1.1)abcfgi
	TAP23C-1327	23.2–28.5 (26.8 ± 1.4)cdehijkl	13.5–16.8 (14.9 ± 0.9)efhjk
	TAP23C-1339	24.8–29.5 (27.3 ± 1.2)cdgh	11.6–16.6 (13.6 ± 1.0)bcd
	TAP23C-1346	24.4–29.3 (26.9 ± 1.1)cdhijl	11.7–16.7 (14.5 ± 0.9)aefgi
	TAP23C-1351	25–30 (26.7 ± 1.3)cdehijkl	12.8–15.6 (14 ± 0.7)abcgi
	TAP23C-1357	24.2–30.2 (27 ± 1.8)cdeghijkl	11.7–15.4 (14.2 ± 0.9)abefgi
	TAP23C-1369	20.8–29.5 (27 ± 2)cdeghijklp	12.3–14.9 (13.9 ± 0.7)abci
	TAP23C-1408	19.3–29.5 (24.8 ± 2.8)abklmp	11.3–15.2 (13 ± 1)d
	TAP24C-0111	24.0–20.3 (26.7 ± 1.3)cdehijkl	13.7–17.6 (15.5 ± 1.0)hjklm
	TAP24C-0116	22.4–30.2 (26.2 ± 2.2)cdehijklmp	12.5–16.6 (14.3 ± 1.1)abefgi
	TAP24C-0119	24.6–31.6 (27.8 ± 1.7)cdfghin	12.3–16.3 (14.2 ± 0.8)abefgi
	TAP25C-0100	23.9–30.5 (27.6 ± 1.6)cdfghi	12.6–15.6 (14.1 ± 0.7)abfgi
*L. brasiliensis*	MAFF306028	19.8–26.7 (23.1 ± 1.5)ab	12.1–16.1 (13.6 ± 0.8)abcd
	TAP23C-1271	24.7–32.9 (28 ± 2)cdfghino	13.0–17.0 (14.5 ± 0.9)aefghi
	TAP23C-1288	14.8–29.2 (25.9 ± 2.7)bcdehijklmp	12.3–25.2 (14.5 ± 2.3)abcdefghijklm
	TAP23C-1289	24.4–29.9 (26.5 ± 1.3)cehijklp	11.6–16.5 (14.2 ± 1.1)abcefgi
	TAP23C-1290	23.5–29.7 (26.8 ± 1.3)cdehijkl	12.6–17.1 (14.7 ± 1.1)efghi
	TAP23C-1328	24.5–34 (29.7 ± 2.7)fno	13.8–17.4 (15.9 ± 0.8)ln
	TAP24C-0019	24.1–29.8 (27.3 ± 1.2)cdgh	12.9–16.1 (14.4 ± 0.7)aefgi
	TAP24C-0103	25.5–30.2 (27.8 ± 1.3)dfg	13.0–15.9 (14.4 ± 0.7)aefgi
	TAP24C-0117	23.8–31.7 (29.1 ± 1.7)fno	13.8–17.8 (15.9 ± 1)lmno
	TAP24C-0118	25.0–30.0 (27.5 ± 1.2)cdgh	12.9–17.2 (15.0 ± 1.0)ehjkm
	TAP24C-0123	24.8–29.9 (27.7 ± 1.2)cdfg	12.4–16.6 (14.3 ± 0.9)abefgi
	TAP24C-0129	23.1–29.8 (26.5 ± 1.6)ehijkl	12.3–16.1 (14.1 ± 0.9)abfgi
	TAP24C-0130	24.5–29.2 (27.1 ± 1.1)cdghi	12.7–16.9 (14.8 ± 0.9)efh
	TAP24C-0143	23.6–30.4 (27.2 ± 1.5)cdghi	13.3–17.4 (15.6 ± 0.9)jklm
	TAP24C-0228	23.7–30.4 (27.4 ± 1.4)cdgh	12.2–16.9 (14.8 ± 1.0)efh
	TAP24C-0525	23.867–29.0 (26.3 ± 1.1)eijkl	13.8–17.2 (15.7 ± 0.8)klm
	TAP24C-0603	26.2–31.5 (29.6 ± 1.6)o	11.6–17.8 (14.6 ± 1.5)abefghijkm
	TAP24C-0031	24.7–31.1 (28.4 ± 1.7)fgno	15.5–17.6 (16.4 ± 0.6)no
	TAP25C-0072	22.4–30.3 (27.4 ± 1.9)cdefghijkl	13.1–17.1 (14.9 ± 1.1)efhjklm
	TAP25C-0095	23–31 (27.9 ± 1.6)cdfghn	12.3–19.2 (14.8 ± 1.3)aefghijklm
	TAP25C-0097	26.6–32.9 (29.5 ± 1.8)no	14.2–19.3 (17.1 ± 1.2)o

^a^ Clades shown on the ML phylogenetic tree in Figure 1. Data with same letters are significantly different (*p* < 0.05).

## Data Availability

Genome sequence data generated in this study will be deposited in a public repository (DBBJ) upon acceptance of the manuscript. Other data supporting the findings of this study are available from the corresponding author upon reasonable request.

## Supplementary Materials

The following supporting information can be downloaded at: https://www.mdpi.com/article/10.3390/jof12040270/s1, Table S1: Primers used in phylogenetic analysis of *Lasiodiplodia* spp.; Table S2: PCR conditions used for each region; Table S3: List of *Lasiodiplodia* spp. and their GenBank accessions used in this study; Table S4: Isolates of *Lasiodiplodia* obtained from different cacao farms in the Philippines. References [8,12,13,16,33,34,35,36,37,38,39,40,41,42,43,44,45] are cited in the Supplementary Materials.

## Abbreviations

The following abbreviations are used in this manuscript:

ITS	internal transcribed spacer
CTAB	cetyltrimethylammonium bromide
PDA	potato dextrose agar
WA	water agar
NJ	neighbor-joining
ML	maximum likelihood
MP	maximum parsimony
ANOVA	analysis of variance
