# Supplementary material for: Genome-Scale Phylogenetic Evidence Supports the Synonymy of Lasiodiplodia brasiliensis with Lasiodiplodia theobromae
Source: J Fungi (Basel). 2026 Apr 8;12(4):270. doi: 10.3390/jof12040270 (PMC13117424; doi:10.3390/jof12040270)
Supplement: Supplementary file 1 [file jof-12-00270-s001.zip › jof-4146597-supplementary.pdf]

Supplementary Table S1. Primers used in phylogenetic analysis of *Lasiodiplodia* spp.

Gene Region	Primer	Direction	Sequence (5′-3′)	Used for	Reference
ITS	ITS-5<br>ITS-4	Forward<br>Reverse	GGAAGTAAAAGTCGTAACAAGG<br>TCCGTAGGTGAACCTGCGG	PCR and Cycle sequence	White et al. (1990) [10]
tub2	Bt2a<br>Bt2b	Forward<br>Reverse	GGTAACCAAATCGGTGCTGCTTTC<br>ACCCTCAGTGTAGTGACCCTTGGC	PCR and Cycle sequence	Glass & Donaldson (1995) [11]
	btLasF<br>btLasR	Forward<br>Reverse	GCGTAAGTCTCCTCCAGCCT<br>TGAGGAGCGTACCCATACCG	PCR and Cycle sequence	This study
rpb2	rpb2-LasF<br>rpb2-LasR	Forward<br>Reverse	GGTAGCGACGTCACTCCT<br>TTTTTGCATCATGAGTTGGAC	PCR and Cycle sequence	Cruywagen et al. (2017) [12]
tef1- α	EF1-LasioF<br>EF1-LasioR	Forward<br>Reverse	GACCACCACCGGTCGTAAGT<br>GGTAACGCTCCTCAGACCAC	PCR and Cycle sequence	This study

Supplementary Table S2. PCR conditions used for each region

Gene Region	Initial Denaturation	Denaturation	Annealing	Extension	Final Extension
ITS	94 °C/3 min	94°C/1 min	55°C/1 min<br>30 cycles	72°C/1 min	72°C/10 min
tub2					
bt2a/bt2b	95°C/5 min	94°C/30 s	60°C/45 s<br>30 cycles	72°C/90 s	72°C/10 min
btLasF/btLasR	95°C/5 min	95°C/30 s	61°C/45 s<br>30 cycles	72°C/1 min	72°C/8 min
rpb2	95°C/2 min	94°C/30 s	64°C/30 s<br>35 cycles	72°C/1 min	72°C/8 min
tef1- α	95°C/5 min	94°C/30 s	61°C/45 s<br>30 cycles	72°C/90 s	72°C/10 min

Supplementary Table S3. List of *Lasiodiplodia* spp. and their GenBank accessions used in this study.

Species	Isolate	Host	Location	GenBank accession number				References
				ITS	*tef1-α*	*tub2*	*rpb2*	
L. acaciae	CBS:136434*	*Acacia* sp.	Indonesia	MT587421	MT592133	MT592613	MT592307	Zhang et al. (2021) [33]
L. aquilariae	CGMCC 3.18471*	*Aquilaria crassna*	Laos	KY783442	KY848600	OM929181	KY848562	Wang, et al. (2019) [34]
	CMW41467*	*Avicennia marina*	South Africa	NR_147359	KP860680	KP860758	KU587878	Osorio et al. (2017) [35]
L. citricola	CBS124707*	*Citrus* sp.	Iran	GU945354	GU945340	KU887505	KU696351	Cruywagen et al (2017) [12] Abdollahzadeh et al. (2010) [36]
L. crassispora	CBS118741*	*Santalum album*	Australia	DQ103550	DQ103557	KU887506	KU696353	Cruywagen et al.[12]
L. curvata	CGMCC 3.18456	*Aquilaria crassna*	Laos	KY783437	KY848596	KY848529	KY848557	Wang, et al. (2019) [34]
L. euphorbiaceicola	CMM3609*	*Jatropha curcas*	Brazil	KF234543	KF226689	KF254926	KU887367	Machado et al. [37]
	CMW33268	*Adansonia* sp.	Unknown	KU887131	KU887008	KU887430	KU887367	Cruywagen et al. (2017) [12]
L. exigua	CMW33323	*Adansonia* sp.	Namibia	KU887200	KU886812	KU887445	KU887377	Cruywagen et al. (2017) [12]
	CMW36172	*Adansonia* sp.	Cameroon	KU887307	KU886916	KU887492	KU887411	Cruywagen et al. (2017) [12]
L. gilanensis	IRAN 1523C*	Unknown	Iran	NR_147328	GU945342	KP872411	KP872462	Abdollahzadeh et al. (2010) [36]
L. gonubiensis	CBS 115812*	*Syzygium cordatum*	South Africa	NR_111218	DQ458877	KU887512	KP872464	Alves et al. (2008) [28]
L. hormozganensis	CBS124708	*Mangifera indica*	Iran	GU945356	GU945344	KU887514	KU696360	Cruywagen et al. (2017) [12], Abdollahzadeh et al. (2010) [36]
	MAFF 240591	*Aloe vera*	Japan	LC567315	LC567744	LC567774	LC567804	Hattori et al. (2023) [31]
L. hormozganensis	MAFF 237946	*Cocos nucifera*	Japan	LC567312	LC567741	LC567771	LC567801	Hattori et al. (2023) [31]
	PH22-014	*Theobroma cacao*	Philippines	LC795569	LC795571	LC795570	LC795572	Ocampo-Padilla et al. (2024) [13]
	CBS124709*	*Olea* sp.	Iran	GU945355	GU945343	KU887515	KU696361	Cruywagen et al. (2017) [12], Abdollahzadeh et al. (2010) [36]
L. hyalina	CGMCC 3.18383	*Acacia confusa*	China	KY767661	KY751302	KY751299	KY751296	Dou et al. (2017) [8]
L. iranensis	CMW33311	*Adansonia* sp.	Madagascar	KU887084	KU886962	KU887442	KU887375	Cruywagen et al. (2017) [12]
	CMW33252	*Adansonia* sp.	Madagascar	KU887065	KU886947	KU887422	KU887360	Cruywagen et al. (2017) [12]
	CBS124710*	*Salvadora persica*	Iran	GU945348	GU945336	KU887516	KU696363	Cruywagen et al. (2017) [12] Abdollahzadeh et al. (2010) [36]
	CBS124711	*Juglans* sp.	Iran	GU945347	GU945335	KU887517	KU696362	Cruywagen et al. (2017) [12], Abdollahzadeh et al. (2010) [36]

Isolates in bold represent strains obtained and sequenced in this study. An asterisk (*) indicates ex-type strains.

Supplementary Table S3. continued.

Species	Isolate	Host	Location	GenBank accession number				References
				ITS	*tef1-α*	*tub2*	*rpb2*	
L. irregularis	CGMCC 3.18468	*Aquilaria crassna*	Laos	KY783472	KY848610	KY848553	KY848592	Wang, et al. (2019) [34]
L. jatrophicola	CBS 111008	*Jatropha curcas*	–	MT587431	MT592143	MT592625	MT592321	Zhang et al., (2021) [33]
L. laeliocattleyae	CBS 167.28*	*Laeliocattleya*	Italy	NR_147364	KU507454	MT592618	MT592313	Rodriguez-Galvez, et al. (2017) [38]
L. laosensis	CGMCC 3.18464*	*Aquilaria crassna*	Laos	NR_182801	KY848609	KY848552	KY848591	Wang et al. (2019) [34]
L. lignicola	CBS134112*	Dead wood	Thailand	JX646797	KU887003	JX646845	KU696364	Cruywagen et al. (2017) [12], Liu et al. (2012) [39]
L. mahajangana	CBS124925*	*Terminalia catappa*	Madagascar	FJ900595	FJ900641	OL405580	KU696365	Cruywagen et al. (2017) [12], Begoude et al. (2010) [40]
	CBS124926	*Terminalia catappa*	Madagascar	FJ900596	FJ900642	KX464902	KU696366	Cruywagen et al. (2017) [12], Begoude et al. (2010) [40]
	CBS 125266	*Terminalia sambesiaca*	South Africa	MT587436	MT592148	KP872409	MT592329	Zhang et al. (2021) [33]
	CBS122519*	*Adansonia gibbosa*	Australia	EU144050	EU144065	KU887520	KU696367	Cruywagen et al. (2017) [12]
	CBS138289	*Combretum elae-agnoides*	Namibia	KP872320	KP872349	KP872379	KP872429	Zhang et al.(2021) [33]
L. mediterranea	CBS137783*	*Quercus ilex*	Italy	NR_147352	KU720487	KU887521	KU696368	Zhang et al. (2021) [33], Linaldeddu et al. (2015) [41]
L. plurivora	STE-U5803	*Prunus salicina*	South Africa	EF445362	EF445395	KP872421	KP872479	Damm et al., (2007) [42]
L. pseudotheobromae	CBS116459*	*Gmelina arborea*	Costa Rica	EF622077	EF622057	EU673111	KU696376	Alves et al. (2008) [28], Phillips et al. (2008) [3]
	CBS116460	*Acacia mangium*	Costa Rica	MT587433	MT592145	KU198428	MT592322	Zhang et al., (2021) [33]
	MAFF 241277	*Rosa* sp.	Japan	LC567327	LC567756	LC567786	LC567816	Hattori et al. (2023) [31]

Isolates in bold represent strains obtained and sequenced in this study. An asterisk (*) indicates ex-type strains.

Supplementary Table S3. (continued)

Species	Isolate	Host	Location	GenBank accession number				References
				ITS	*tef1-α*	*tub2*	*rpb2*	
L. pseudotheobromae	PH22-080	*Theobroma cacao*	Philippines	LC795577	LC795579	LC795578	LC795580	Ocampo-Padilla et al. (2024) [13]
L. rubropurpurea	WAC 12535*	*Eucalyptus grandis*	Australia	NR_136976.1	EU673304	KU887529	KU696380	Burgess, et al. (2006) [43]
L. sterculiae	CBS:342.78	*Sterculia oblonga*	Germany	KX464140	KX464634	KX464908	KX463989	Yang et al., (2017) [44]
L. thailandica	MAFF 244514	*Brophyllum pinnatum*	Japan	LC567321	LC567750	LC567780	LC567810	Hatorri et al (2023) [31]
L. theobromae	PH22-0007	*Theobroma cacao*	Philippines	LC795565	LC795567	LC795566	LC795568	Ocampo-Padilla et al. (2024) [13]
	PH22-0120	*Theobroma cacao*	Philippines	LC795581	LC795583	LC795582	LC795584	Ocampo-Padilla et al. (2024) [13]
	**TAP23C-1267**	***Theobroma cacao***	**Philippines**	**LC923723**	**LC923840**	**LC923762**	**LC923801**	**This study**
	**TAP23C-1269**	***Theobroma cacao***	**Philippines**	**LC923724**	**LC923841**	**LC923763**	**LC923802**	**This study**
	**TAP23C-1278**	***Theobroma cacao***	**Philippines**	**LC923725**	**LC923842**	**LC923764**	**LC923803**	**This study**
	**TAP23C-1302**	***Theobroma cacao***	**Philippines**	**LC923726**	**LC923843**	**LC923765**	**LC923804**	**This study**
	**TAP23C-1303**	***Theobroma cacao***	**Philippines**	**LC923727**	**LC923844**	**LC923766**	**LC923805**	**This study**
	**TAP23C-1312**	***Theobroma cacao***	**Philippines**	**LC923728**	**LC923845**	**LC923767**	**LC923806**	**This study**
	**TAP23C-1317**	***Theobroma cacao***	**Philippines**	**LC923729**	**LC923846**	**LC923768**	**LC923807**	**This study**
	**TAP23C-1324**	***Theobroma cacao***	**Philippines**	**LC923730**	**LC923847**	**LC923769**	**LC923808**	**This study**
	**TAP23C-1327**	***Theobroma cacao***	**Philippines**	**LC923731**	**LC923848**	**LC923770**	**LC923809**	**This study**
	**TAP23C-1339**	***Theobroma cacao***	**Philippines**	**LC923732**	**LC923849**	**LC923771**	**LC923810**	**This study**
	**TAP23C-1346**	***Theobroma cacao***	**Philippines**	**LC923733**	**LC923850**	**LC923772**	**LC923811**	**This study**
	**TAP23C-1351**	***Theobroma cacao***	**Philippines**	**LC923734**	**LC923851**	**LC923773**	**LC923812**	**This study**
	**TAP23C-1357**	***Theobroma cacao***	**Philippines**	**LC923735**	**LC923852**	**LC923774**	**LC923813**	**This study**
	**TAP23C-1369**	***Theobroma cacao***	**Philippines**	**LC923736**	**LC923853**	**LC923775**	**LC923814**	**This study**
	**TAP23C-1408**	***Theobroma cacao***	**Philippines**	**LC923737**	**LC923854**	**LC923776**	**LC923815**	**This study**
	**TAP24C-0111**	***Theobroma cacao***	**Philippines**	**LC923738**	**LC923855**	**LC923777**	**LC923816**	**This study**
	**TAP24C-0116**	***Theobroma cacao***	**Philippines**	**LC923739**	**LC923856**	**LC923778**	**LC923817**	**This study**

Isolates in bold represent strains obtained and sequenced in this study. An asterisk (*) indicates ex-type strains.

Supplementary Table S3. (continued)

Species	Isolate	Host	Location	GenBank accession number				References
				ITS	*tef1-α*	*tub2*	*rpb2*	
L. theobromae	**TAP24C-0119**	***Theobroma cacao***	**Philippines**	**LC923740**	**LC923857**	**LC923779**	**LC923818**	**This study**
	**TAP25C-0100**	***Theobroma cacao***	**Philippines**	**LC923741**	**LC923858**	**LC923780**	**LC923819**	**This study**
	CBS214.50	*Cajanus cajan*	India	MT587440	MT592152	MT592637	MT592333	Zhang et al. (2021) [33]
	NS2F	*Theobroma cacao*	Malaysia	OL831055	OL863319	OL863262	OL863376	Huda-Shakira et al. (2022) [16]
	MAFF 243205	*Theobroma cacao*	Japan	LC567301	LC567730	LC567760	LC567790	Hattori et al. (2023) [31]
	MAFF 306028	*Annona squamosa*	Japan	LC567306	LC567735	LC567765	LC567795	Hattori et al. (2023) [31]
	CBS164.69*	Fruit on coral reef coast	Indonesia	AY640255	AY640258	EU673110	KU696383	Cruywagen et al. (2017) [12]
L. theobromae (syn. *L. brasiliensis*)	CBS120395	*Theobroma cacao*	Cameroon	MT587423	MT592135	MT592615	MT592309	Zhang et al. (2021) [33]
	MUCC 2553	*Mangifera indica*	Japan	LC567299	LC567728	LC567758	LC567788	Hatorri et al. (2023) [31]
	MUCC 2729	*Mangifera indica*	Japan	LC567300	LC567729	LC567759	LC567789	Hatorri et al. (2023) [31]
	CBS 115447	*Psychotria tutcheri*	Hong Kong	MT587422	MT592134	MT592614	MT592308	Zhang et al. (2021) [31]
	**TAP23C- 1271**	***Theobroma cacao***	**Philippines**	**LC923742**	**LC923859**	**LC923781**	**LC923820**	**This study**
	**TAP23C- 1288**	***Theobroma cacao***	**Philippines**	**LC923743**	**LC923860**	**LC923782**	**LC923821**	**This study**
	**TAP23C- 1289**	***Theobroma cacao***	**Philippines**	**LC923744**	**LC923861**	**LC923783**	**LC923822**	**This study**
	**TAP23C- 1290**	***Theobroma cacao***	**Philippines**	**LC923745**	**LC923862**	**LC923784**	**LC923823**	**This study**
	**TAP23C- 1328**	***Theobroma cacao***	**Philippines**	**LC923746**	**LC923863**	**LC923785**	**LC923824**	**This study**
	**TAP24C-0525**	***Theobroma cacao***	**Philippines**	**LC923756**	**LC923873**	**LC923795**	**LC923834**	**This study**
	**TAP24C-0603**	***Theobroma cacao***	**Philippines**	**LC923757**	**LC923874**	**LC923796**	**LC923835**	**This study**
	**TAP25C-0228**	***Theobroma cacao***	**Philippines**	**LC923755**	**LC923872**	**LC923794**	**LC923833**	**This study**
	**TAP24C-0019**	***Theobroma cacao***	**Philippines**	**LC923747**	**LC923864**	**LC923786**	**LC923825**	**This study**
	**TAP25C-0031**	***Theobroma cacao***	**Philippines**	**LC923758**	**LC923875**	**LC923797**	**LC923836**	**This study**
	**TAP25C-0095**	***Theobroma cacao***	**Philippines**	**LC923760**	**LC923877**	**LC923799**	**LC923838**	**This study**
	**TAP25C-0097**	***Theobroma cacao***	**Philippines**	**LC923761**	**LC923878**	**LC923800**	**LC923839**	**This study**

Isolates in bold represent strains obtained and sequenced in this study. An asterisk (*) indicates ex-type strains.

Supplementary Table S3. (continued)

Species	Isolate	Host	Location	GenBank accession number				References
				ITS	***tef1-α***	***tub2***	***rpb2***	
L. theobromae (syn. *L. brasiliensis*)	**TAP24C-0103**	***Theobroma cacao***	**Philippines**	**LC923748**	**LC923865**	**LC923787**	**LC923826**	**This study**
	**TAP24C-0117**	***Theobroma cacao***	**Philippines**	**LC923749**	**LC923866**	**LC923788**	**LC923827**	**This study**
	**TAP24C-0118**	***Theobroma cacao***	**Philippines**	**LC923750**	**LC923867**	**LC923789**	**LC923828**	**This study**
	**TAP24C-0123**	***Theobroma cacao***	**Philippines**	**LC923751**	**LC923868**	**LC923790**	**LC923829**	**This study**
	**TAP24C-0129**	***Theobroma cacao***	**Philippines**	**LC923752**	**LC923869**	**LC923791**	**LC923830**	**This study**
	**TAP24C-0130**	***Theobroma cacao***	**Philippines**	**LC923753**	**LC923870**	**LC923792**	**LC923831**	**This study**
	**TAP25C-0072**	***Theobroma cacao***	**Philippines**	**LC923759**	**LC923876**	**LC923798**	**LC923837**	**This study**
	**TAP24C-0143**	***Theobroma cacao***	**Philippines**	**LC923754**	**LC923871**	**LC923793**	**LC923832**	**This study**
L. tropica	CGMCC 3.18477	*Aquilaria crassna*	Laos	KY783454	KY848616	KY848540	KY848574	Wang et al. (2019) [34]
L. viticola	CBS128313*	hybrid grape Vignoles	USA	HQ288227	HQ288269	HQ288306	KU696385	Cruywagen et al. (2017) [12]
L. vitis	CBS 124060	*Vitis vinifera*	Italy	KX464148	KX464642	KX464917	KX463994	Yang et al., (2017) [44]
Lasiodiplodia sp.	MAFF 411002	*Laburnum anagyroides*	Japan	LC567324	LC567753	LC567783	LC567813	Hattori et al. (2023) [31]
	MAFF 242322	*Phoenix roebelenii*	Japan	LC567328	LC567757	LC567787	LC567817	Hattori et al. (2023) [31]
Lasiodiplodia sp.	TAP22C-0060	*Theobroma cacao*	Philippines	LC795573	LC795575	LC795574	LC795576	Ocampo-Padilla et al. (2024) [13]
Botryospaheria dothidea	CBS 115476	*Prunus* sp.	Switzerland	KF766151	AY236898	MT592470	DQ677944	Slippers et al. (2013) [45]

Isolates in bold represent strains obtained and sequenced in this study. An asterisk (*) indicates ex-type strains.

Supplementary Table S4. Isolates of *Lasiodiplodia* obtained from different cacao farms in the Philippines

Species name	Strain	Substrate	Location
L. theobromae	PH22-007	Leaf litter	Manuel Quezon, Nueva Ecija
	TAP23C-1267	Symp leaf	Pozzorubio, Pangasinan
	TAP23C-1269	Symp leaf	Pozzorubio, Pangasinan
	TAP23C-1278	Symp leaf	Pozzorubio, Pangasinan
	TAP23C-1351	Asymp flower	Pozzorubio, Pangasinan
	TAP23C-1357	Asymp flower	Pozzorubio, Pangasinan
	TAP23C-1327	Asymp flower	Pozzorubio, Pangasinan
	TAP23C-1324	Symp flower	Pozzorubio, Pangasinan
	TAP23C-1339	Symp flower	Pozzorubio, Pangasinan
	TAP23C-1346	Symp flower	Pozzorubio, Pangasinan
	TAP23C-1369	Symp flower	Pozzorubio, Pangasinan
	TAP23C-1302	Symp stem	Pozzorubio, Pangasinan
	TAP23C-1303	Symp stem	Pozzorubio, Pangasinan
	TAP23C-1312	Symp stem	Pozzorubio, Pangasinan
	TAP23C-1317	Symp stem	Pozzorubio, Pangasinan
	TAP24C-0111	Symp stem	Tagakpan, Davao City
	TAP24C-0116	Symp stem	Tagakpan, Davao City
	TAP24C-0119	Symp stem	Tagakpan, Davao City
	TAP25C-0100	Symp stem	Calinan, Davao City
	PH22-120	Symp pod	San Jacinto, Pangasinan
	TAP23C-1408	Symp pod	Pozzorubio, Pangasinan
L. theobromae (syn. *L. brasiliensis*)	TAP23C-1271	Symp leaf	Pozzorubio, Pangasinan
	TAP23C-1288	Symp leaf	Pozzorubio, Pangasinan
	TAP23C-1289	Symp leaf	Pozzorubio, Pangasinan
	TAP23C-1290	Symp leaf	Pozzorubio, Pangasinan
	TAP24C-0228	Symp leaf	Calinan, Davao City
	TAP24C-0525	Symp leaf	Calinan, Davao City
	TAP24C-0603	Symp leaf	Calinan, Davao City
	TAP24C-0031	Symp leaf	Tagakpan, Davao City
	TAP24C-0019	Symp leaf	Tagakpan, Davao City
	TAP23C-1328	Asymp flower	Pozzorubio, Pangasinan
	TAP24C-0103	Symp stem	Tagakpan, Davao City
	TAP24C-0117	Symp stem	Tagakpan, Davao City
	TAP24C-0118	Symp stem	Tagakpan, Davao City
	TAP24C-0123	Symp stem	Tagakpan, Davao City
	TAP24C-0129	Symp stem	Tagakpan, Davao City
	TAP24C-0130	Symp stem	Tagakpan, Davao City
	TAP25C-0072	Symp stem	Tagakpan, Davao City
	TAP25C-0095	Symp stem	Mintal, Davao City
	TAP25C-0097	Symp stem	Mintal, Davao City
	TAP24C-0143	Symp pod	Tagakpan, Davao City

Symp- refers to symptomatic; Asymp- refers to asymptomatic
